# Hôpital Beaujon

**Published:** 1833-01

**Authors:** 


### HOPITAL BEAUJON.

*Case of Fungus in the Bladder, reported by* Dr. Nicod, Surgeon in Chief of the Hôpital Beaujon.

M. Fasquel, sixty-four years of age, a large and robust man, and of a sanguine temperament, who had travelled much and ridden hard on horseback, had for many years been troubled with piles. Finding himself one day more inconvenienced with the hemorrhoidal tumors than usual, he had recourse to a cold hip-bath, from which he experienced great relief: in fact, the piles disappeared; but for several years afterwards he was occasionally afflicted with slight hæmaturia. This new symptom recurred from time to time, and was aggravated whenever he travelled over paved roads or journeyed on horseback. At the beginning of the year 1826, the affection had so much increased, that he could not pursue his accustomed journeys, even for a day, without losing a considerable quantity of blood, which, from its increase in quantity, became more and more alarming.

On the 19th of December, he consulted M. Nicod, and informed him that, notwithstanding every precaution in travelling over the unpaved parts of the roads, he had, on his way to him, passed about two cupfuls of blood from the bladder; that he suffered with continual pain at the glans penis; that his urine was at times clear, at others of a dark red hue, and occasionally with a mucous sediment: sometimes it passed freely, and at others with difficulty.

The bladder was examined by the catheter, which was introduced with tolerable ease, and brought away small fibrinous fragments; and, on repeating the introduction with a silver instrument, which was arrested in its progress at about two inches from the neck of the bladder, several fleshy and membranous shreds came away; and, when he passed his urine, it was at first mixed with blood, but subsequently became limpid.

On the morrow, the exploration was renewed with the same instruments, which brought away still more debris; and the patient passed a little blood during the afternoon. On the third day, he passed water more easily and frequently than heretofore, which encouraged him to persevere in the plan of treatment proposed.

Very frequently during the current week, the urethra became partially clogged up with fragments of a fungous or fleshy matter, which, according to the degree of force employed, were detached in larger or smaller shreds from the mass against which the instrument was pressed; and which, when the bladder was emptied of water, seemed to be about the size of the closed fist, and nearly to fill the whole of the vesical cavity. The softness of the tumor, which the examination rendered evident, considered along with the absence of sabulous deposits in the urine, led M. Nicod to determine this extraordinary malady was not complicated with stone in the bladder, and that it consisted of a fungus only.

Encouraged by the visible improvement of the patient's health, and the readiness with which the hemorrhage disappeared, after having passed the catheter four or five times, the operation was repeated twice a day, or more, according to circumstances.

Frequently portions of transparent membrane came away, having a resemblance to fragments of nasal polypi; one of which, when floated in water, was something of the shape of the blossom of a convolvulus, and at least two inches across.

Every day the facility of introducing and moving the instrument in the bladder increased, so that subsequent catheterism proved that no stone existed in the viscus, but that the diagnosis had been correct; the tumor consisting of a fungus only, which was attached on the right side of the *bas fond* of the bladder. The more the fungus was destroyed, the more solid did the remaining part become; and, the more the eyes of the instrument were pressed against it, the more pure blood was voided. The bladder, however, had lost much of the morbid sensibility it exhibited during the early stages of the treatment: this led to the frictions on the tumor being increased, and which terminated by the entire destruction even of the pedicle, which was about eighteen lines in diameter from before backwards. The diseased spot gradually shrunk, and at last ceased to yield any more blood to reiterated frictions by the catheter.

The patient's appetite improved, and week after week his strength increased, and, at the end of about six weeks, he was enabled to ride round Boulogne wood, in a carriage, without passing a drop of blood; and, some days after, he went to Versailles and back, only stopping there an hour, without suffering any hemorrhage. In seven weeks he was entirely cured; and remained well until the year 1831, when he died, at the age of sixty-nine, of apoplexy, attended with a chronic disease of the liver.

This case has proved, as it were, almost by chance, the practicability of what Bichat thought scarcely possible, viz. the cure of fungus in the bladder; because, as he says, it is so difficult to arrive at any certain diagnosis of these lamentable diseases; and, consequently, to institute any efficacious means of cure; but this case shows that both are possible, because both were done.—*Gazette Medicale.*

